# A murine model to study vasoreactivity and intravascular flow in lung isograft microvessels

**DOI:** 10.1038/s41598-019-41590-7

**Published:** 2019-03-26

**Authors:** Nora Regelin, Susanne Heyder, Matthias W. Laschke, Yalda Hadizamani, Michèle Borgmann, Ueli Moehrlen, René Schramm, Robert Bals, Michael D. Menger, Jürg Hamacher

**Affiliations:** 1 Department of Internal Medicine V - Pulmonology, Allergology, Respiratory Intensive Care Medicine, Saarland University Hospital, 66424 Homburg, Germany; 2 Institute for Clinical & Experimental Surgery, Faculty of Medicine, Saarland University, 66421 Homburg, Germany; 3 Heart and Diabetes Centre North Rhine-Westphalia, University Hospital of the Ruhr University of Bochum, 32545 Bad Oeynhausen, Germany; 4 Mediclin Albert Schweitzer Clinic, Pneumology, 78126 Königsfeld, Germany; 5 Pneumology, Clinic for General Internal Medicine, Lindenhofspital Bern, 3012 Bern, Switzerland; 6 Lungen-und Atmungsstiftung, Bern, 3012 Bern, Switzerland; 7 Pediatric Surgery, University Children’s Hospital Zurich, 8032 Zurich, Switzerland

## Abstract

Intravital microscopy of orthotopic lung tissue is technically demanding, especially for repeated investigations. Therefore, we have established a novel approach, which allows non-invasive repetitive *in vivo* microscopy of ectopic lung tissue in dorsal skinfold chambers. Syngeneic subpleural peripheral lung tissue and autologous endometrium (control) were transplanted onto the striated muscle within dorsal skinfold chambers of C57BL/6 mice. Grafts were analysed by intravital fluorescence microscopy over 14 days. Angiogenesis occurred in the grafts on day 3, as indicated by sinusoidal microvessels on the grafts’ edges with very slow blood flow, perifocal oedema, and haemorrhage. By day 10, lung transplants were completely revascularized, exhibited a dense network of microvessels with irregular diameters, chaotic angioarchitecture, and high blood flow. Compared to lung tissue, endometrial grafts contained a structured, glomerulus-like vessel architecture with lower blood flow. Despite missing ventilation, hypoxic vasoconstriction of the lung tissue arterioles occurred. In contrast, endometrium tissue arterioles dilated during hypoxia and constricted in hyperoxia. This demonstrates that ectopic lung grafts keep their ability for organ-specific hypoxic vasoconstriction. These findings indicate that our approach is suitable for repetitive *in vivo* pulmonary microcirculation analyses. The high blood flow and hypoxia-induced vasoconstriction in lung grafts suggest a physiological intrinsic vasoregulation independent of the recipient tissue.

## Introduction

Since more than three decades *in vivo* microscopy of organs has allowed to assess tissue morphology at the architectural and cellular level^1–3^. It provides detailed insights into processes of cell movement, adhesion or fluid shifts like extravasation. This technology has entered clinical practice in fields like ophthalmology, dermatology, cardiology, and gastroenterology. In research and clinical settings, *in vivo* microscopy aims to replace invasive procedures with minimally invasive or non-invasive ones^2,4–16^.

Observation chambers like skinfold chambers of hamsters, mice, and rats allow prolonged studies with repeated analyses of the microcirculation over a few weeks^1^, with the experimental and ethical advantage that the animals show a normal behaviour during this period. Dorsal skinfold chambers have thus proved to be versatile to study microcirculation physiology, inflammation and sepsis, ischemia-reperfusion, angiogenesis, and transplantation^1^. Thereby, rats and in particular mice offer the advantage of a huge availability of species-specific tools^17^.

*In vivo* microscopy of orthotopic lung tissue bears several advantages. Respiratory physiology, i.e. pulmonary ventilation, negative intrathoracic pressure and physiological lung blood flow, can chiefly be maintained. Recently, Kübler and Tabuchi have introduced a short-living thorax window model in mice to assess the lung for orthotopic *in vivo* microscopy under physiological conditions, i.e. mainly intrathoracic negative pressure^17–19^. However, such a model works only for few hours, impeding the possibility to study physiological responses over many days. Other difficulties are the minimization of tissue trauma during preparation and three-dimensional movement that may cause analytic problems especially due to physiological or mechanical breathing movements during measurements.

To overcome a part of these problems, we herein present a novel approach, which uses the dorsal skinfold chamber as the host site for pulmonary tissue transplantation. This approach allows for repetitive studies of the grafts’ revascularization and microcirculation over 2–3 weeks as well as leukocyte-endothelial interactions under various conditions.

In a first set of experiments, we analysed the revascularization of the ectopic lung grafts. This process was compared to the revascularization of endometrial tissue, which served as a control tissue, because it has been previously investigated in the dorsal skinfold chamber in detail^20–24^. In a second set of experiments, we analysed the vasoreactivity of microvessels at different hypoxic or hyperoxic inspiratory oxygen fractions to assess whether the two transplanted tissue types keep their typical oxygen-dependent vasoregulatory properties at an ectopic site.

## Results

### Graft revascularization

**Figure 1:**
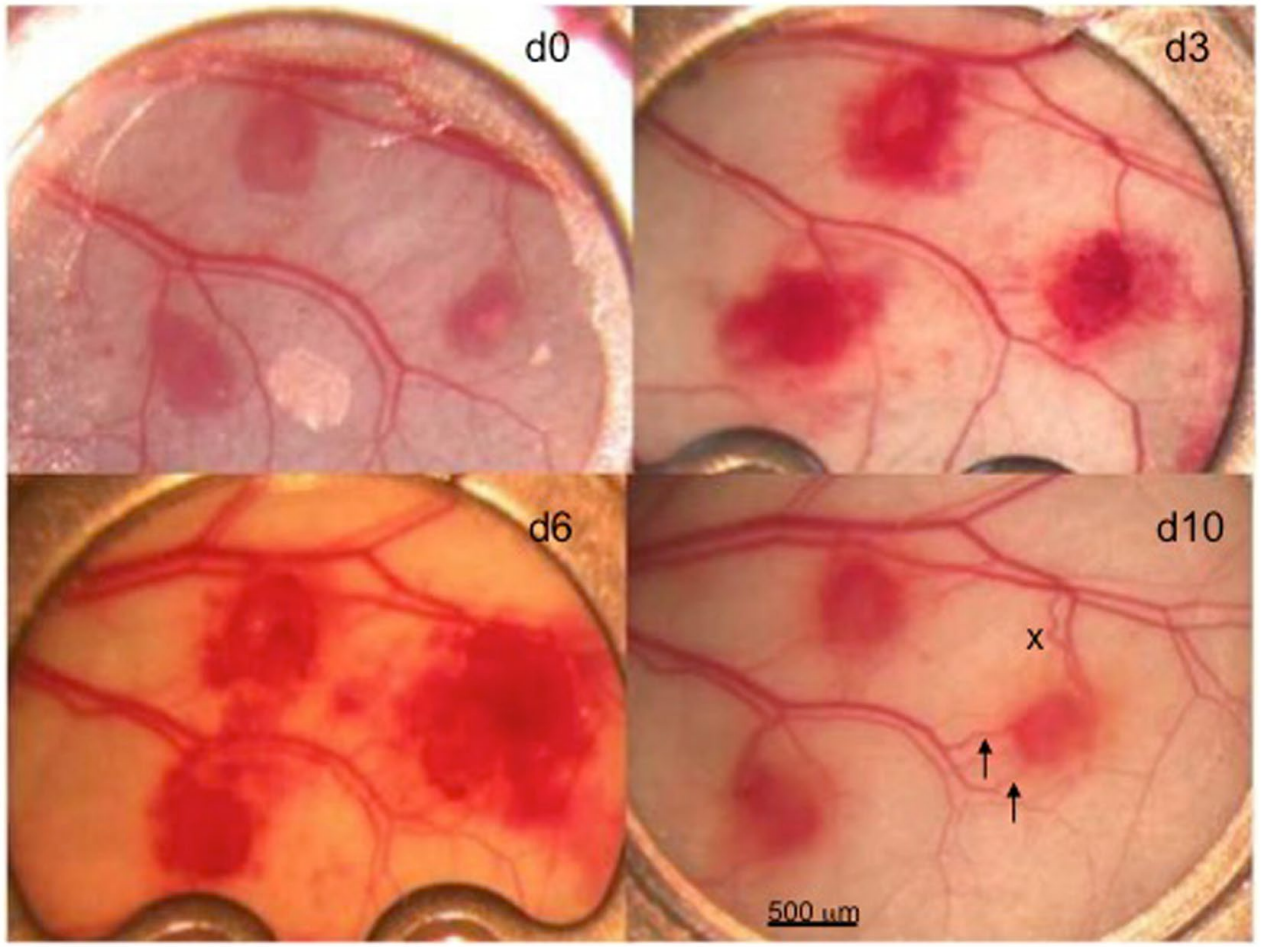
Three lung transplant blocks of about 0.5 mm within the skinfold chamber over time (days 0, 3, 6, and 10). On day 3 there were sinusoidally widened vessels found at the transplant margins with very slow blood flow, a perifocal oedema, and multiple perisinusoidal haemorrhages. Note the recruited vessels around the ectopic lung tissues (e.g. the right-sided lung tissue specimen) that increase over time (→) in diameter and in part even reverse flow (x).

**Figure 2:**
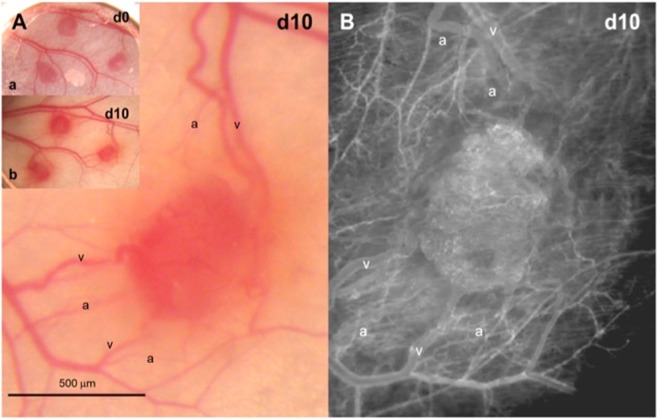
Detail of day 10, right-sided transplant, from Fig. 2. (**A**) Normal intravital epiillumination microscopy of a revascularized lung tissue graft on day 10 after transplantation into the dorsal skinfold chamber of a C57BL/6 mouse: arterioles (a), capillaries (c) and venules (v). (**B**) Intravital fluorescence microscopy of the same revascularized lung tissue graft on day 10 after transplantation into the dorsal skinfold chamber of a C57BL/6 mouse. The corresponding vessels as described in A are seen.

**Figure 3:**
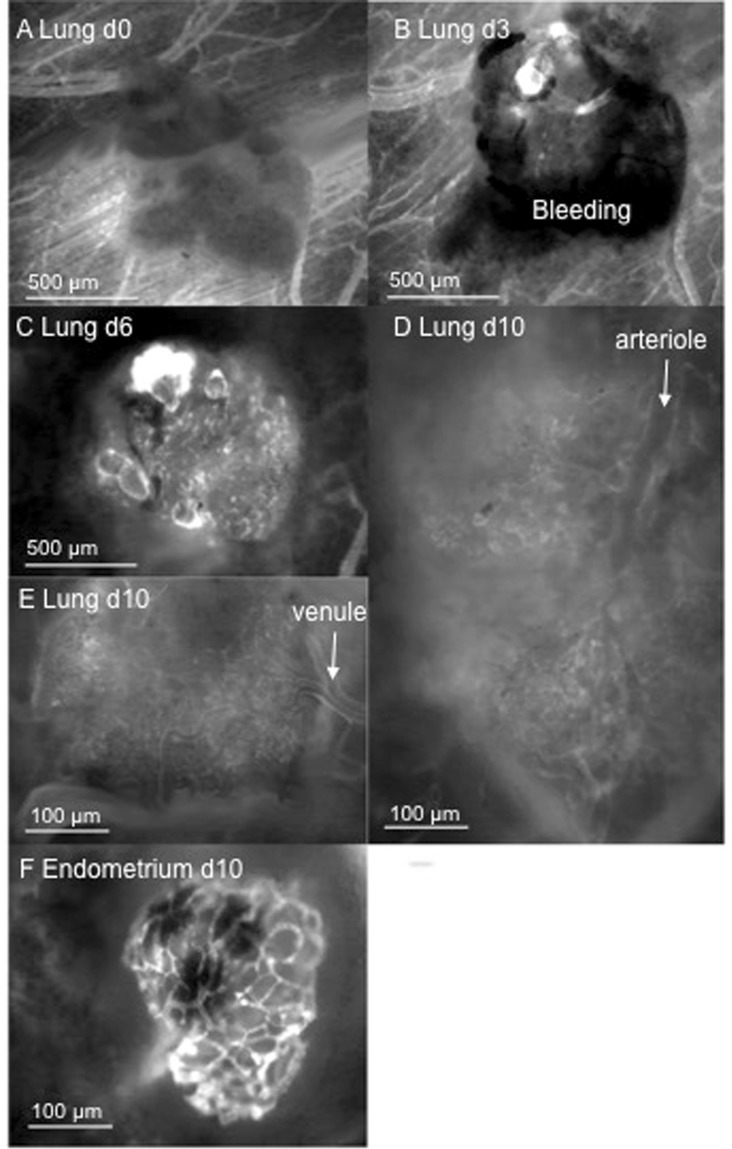
Intravital fluorescence microscopic skinfold chamber images. (**A**) Directly after transplantation of lung tissue into the chamber. (**B**) Lung tissue on day 3: the first sinusoidal vessel connections are seen at the transplant margins, and extravasates are visible. (**C**) Visibility of vessels increases much compared to day 3, pointing at an almost complete vascular embedding of the lung tissue graft. Vessels are seen to be of differing diameters, and sinusoidal vessels are seen mainly at the margins. (**D**,**E**) Totally branched spongiform vessel structures seen on day 10. (**F**) Demonstration of arteriole leading to microvasculature. (**E**) Demonstration of a venule fed by the alveolar microvessels. (**F**) Endometrium on day 10. Compared to lung tissue, the microvascular network seems more glomerulus-like with less branching or longer microvessels leading to branches and seems therefore to be much less branched.

Lung grafts were able to induce angiogenesis and to completely revascularize after transplantation into the dorsal skinfold chamber of recipient mice (Figs 1, 2 and 3). Angiogenesis could be observed on day 3 after transplantation and was characterized by sinusoidal vascular sacculations in the grafts’ margins as well as haemorrhage formation in the grafts and the surrounding tissue (Figs 1 and 3).

**Figure 4:**
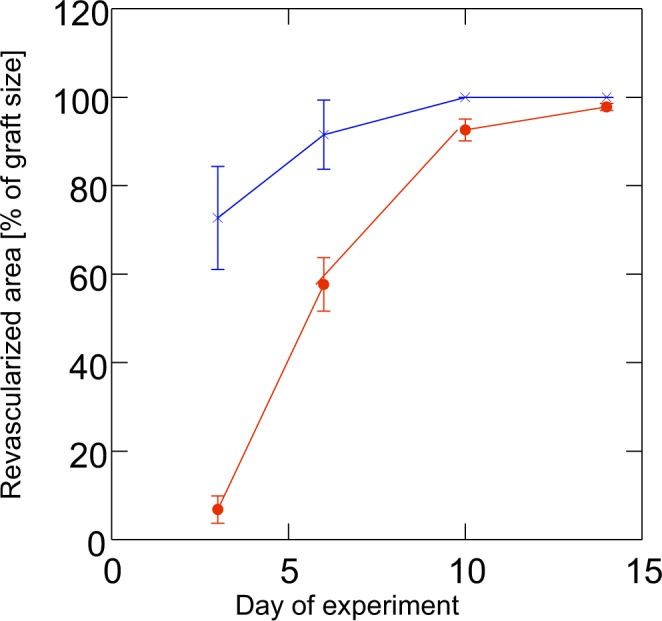
Revascularized area (% of graft size; mean and SEM; n = 10 endometrium grafts out of 7 mice and n = 34 lung grafts out of 15 mice) of lung transplants (filled circles) and of endometrium transplants (x). Note that the revascularization is much faster in endometrium tissue than within lung tissue. Also, haemorrhage may account for areas not counted as revascularized (ANOVA: p < 0.0005 for differences between days, and p < 0.0005 for tissue type).

**Figure 5:**
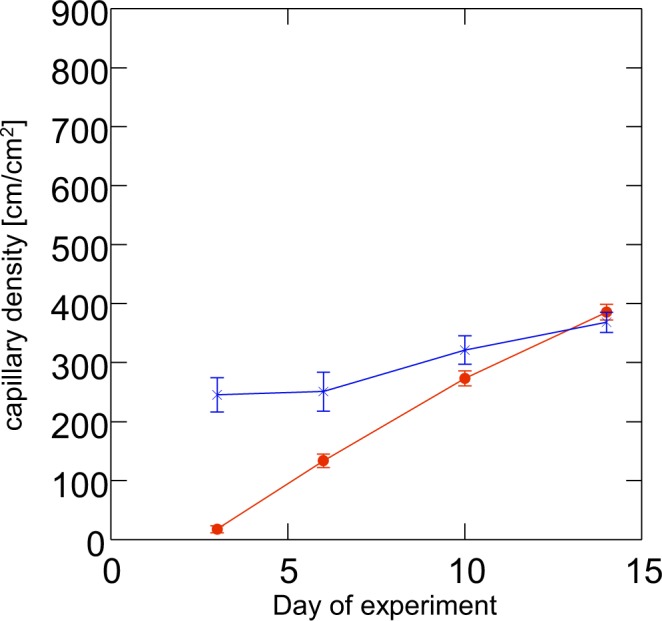
Functional capillary density (mean and SEM; n = 10 endometrium grafts out of 7 mice and n = 34 lung grafts out of 15 mice) of lung transplants (filled circles) and of endometrium transplants (x). Note that the functional capillary density is related to perfuse vasculature; stasis in vessels is not counted. On day 14, endometrium and lung reach about the same capillary density. (ANOVA: p < 0.0005 for differences between days, and p < 0.0005 for tissue.

During the following days, a complete blood-perfused microvascular network developed (Figs 3 and 4). This network exhibited a functional capillary density of ~250–450 cm/cm² (Fig. 5), which was comparable to that of the endometrium transplants, as already described^20^. The initial sinusoidal vessels developed into those newly formed vessels that connected the skinfold chamber tissue vessels to the grafts.

**Figure 6:**
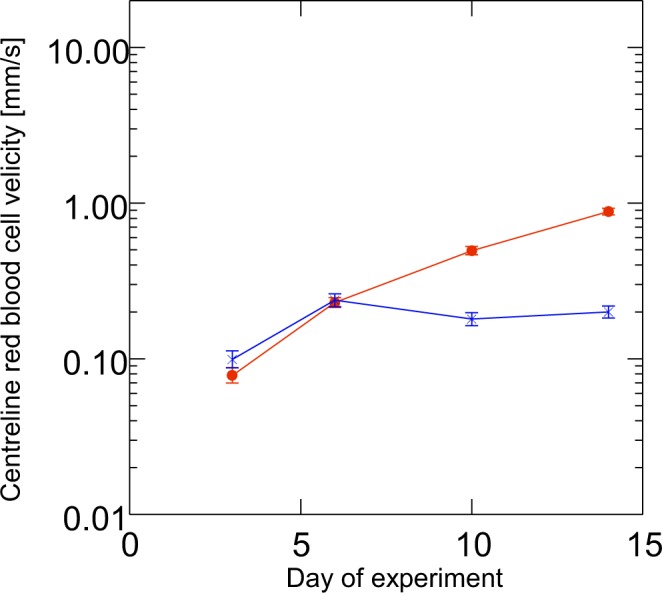
Microvascular centreline RBC velocity of lung (filled circles) and endometrium (x) transplants in dorsal skinfold chambers (mean and SEM; n = 10 endometrium grafts out of 7 mice and n = 34 lung grafts out of 15 mice). Note the increasing centreline RBC velocity over time of the lung vasculature within the 14 days of observation, which reached about 5 to 10-fold the value of endometrium. As mentioned in the text, the centreline RBC velocity has been difficult to assess due to technical reasons and may even be underestimated in lung tissue. Contrary to that, the centreline RBC velocity in endometrium reached its maximal value at 6 days and then remained stable over time.

The centreline red bood cell (RBC) velocity in lung graft vessels progressively increased throughout the observation period to a mean of 1.3 mm/s (Fig. 6). Rhodamine 6G-stained leukocytes showed that almost all leukocytes passed the newly formed microvascular networks without any transient tethering or rolling interactions. Moreover, adherent leukocytes could only rarely be observed, indicating only a low level of leukocytic inflammation in this model.

There was no significant change in the graft size over time.

**Figure 7:**
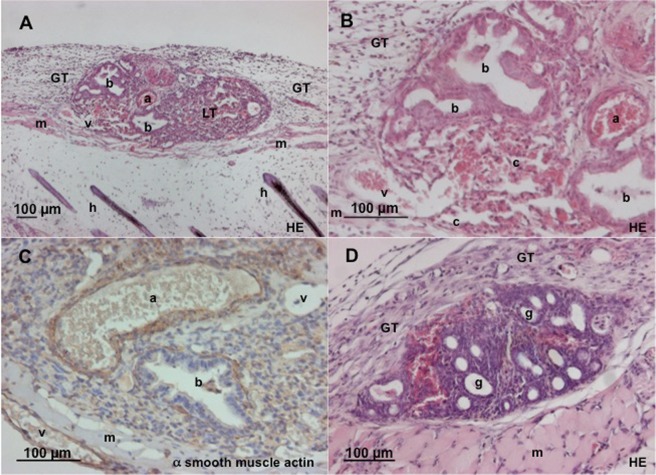
Histological analysis of skinfold chamber with transplants. a = arteriole, v = venule, b = bronchus, c = capillary, h = hair, m = skin muscle, GT = granulation tissue, LT = lung tissue, g = cyst like endometrium glands. (**A**,**B**) HE staining of lung tissue on day 14 after transplantation into the dorsal skinfold chamber of a C57BL/6 mouse. Note that the original vessels of the lung tissue, including arterioles, capillaries and venules, are reperfused. (**C**) α-SMA staining of lung tissue on day 14 after transplantation into the dorsal skinfold chamber. The arteriole can be distinguished from the venule by its strong wall containing smooth muscle actin positive cells. (**D**) HE stain of endometrium tissue on day 14 after transplantation into the dorsal skinfold chamber. Typical cyst-like endometrium glands with richly vascularized stroma can be seen.

Although the angioarchitecture within the grafts was chaotic, it was possible to differentiate between arterioles, capillaries, and venules by identifying blood flow collection versus distribution patterns (Fig. 3). The physiological lung architecture with bronchioles and pulmonary arteries containing RBCs also appeared unchanged in the histological preparations (Fig. 7).

### Histological analysis

In the histological specimens, we further observed that the grafts were covered by some granulation tissue (Fig. 7). There was minimal or no granulation tissue between the grafts and the striated skin muscle. Large connecting vessels were found particularly at the base as well as at the edges of the grafts and seemed to lead to central graft parts. Vessel diameters were observed up to about 70 µm within lung tissues. There was virtually an absence of any inflammatory infiltrates within the grafts, which is concordant to the observation that there were very few adherent leukocytes during *in vivo* microscopy. Well-preserved bronchioli with cilia and sometimes also mucous secretions within the bronchiolar lumina virtually without inflammatory cells were observed (Fig. 7).

Caspase-3-staining of the histologic preparations at day 14 showed no significant cell death in the lung grafts. The median count of apoptotic cells per high power field was 0 in lung and endometrium tissue as well as in chamber tissue.

Alpha-smooth muscle actin (α-SMA) immunostaining confirmed the smooth muscle layers around bronchioles and accompanying arterioles (Fig. 7). Much fewer α-SMA-positive cells were found around venules as well as within the alveolar septal space.

### Comparison of revascularization of lung and endometrium grafts

To compare the revascularization pattern and microvascular properties of lung grafts with that of another tissue, we additionally performed transplantation of autologous endometrium grafts and syngeneic lung tissue grafts in the same skinfold chamber. In contrast to lung grafts with their high bleeding tendency, endometrium grafts seemed to induce less bleeding and revascularized earlier (by day 6 after transplantation). They exhibited microvascular networks with a characteristic glomerulus-like pattern (Fig. 3). At all observation points, there were no sinusoidal structures in or around the endometrium grafts. The vessel diameters as well as maximum RBC velocities after complete revascularization were considerably lower when compared to lung grafts (Table 1, Fig. 6). Histological sections on day 14 after transplantation showed typical cyst-like endometrial glands, which were surrounded by a densely vascularized stroma (Fig. 7). Contrary to lung tissue where large perigraft vessels were found, histological sections of endometrium grafts did not show such vessels at the basis or the edges of the grafts.

**Table 1:** Vessel diameters, centreline RBC velocity and adherent leukocytes in lung and endometrium grafts over time.

day after transplantation	vessel diameter (µm) mean and SEM		Centreline red blood cell velocity (mm/s) mean and SEM		adherent leukocytes/functional capillary density (cells/cm) mean and SEM	
	lung	endometrium	lung	endometrium	Lung	endometrium
d3	31.40 (4.70)	14.40 (0.50)	0.11 (0.02)	0.33 (0.18)		
d6	36.80 (2.30)	14.70 (0.50)	0.43 (0.03)	0.39 (0.05)		
d10	20.40 (0.80)	13.40 (0.30)	0.90 (0.06)	0.29 (0.03)	0.12 (0.01)	0.04 (0.01)
d14	16.90 (0.40)	13.50 (0.30)	1.32 (0.07)	0.28 (0.02)	0.10 (0.01)	0.03 (0.00)

N = 10 endometrium grafts out of 7 mice and 34 lung grafts out of 15 mice.

### Measurements of vessel diameters during hypoxia (FiO_2_ 10%), normoxia and hyperoxia (FiO_2_ 100%)

**Figure 8:**
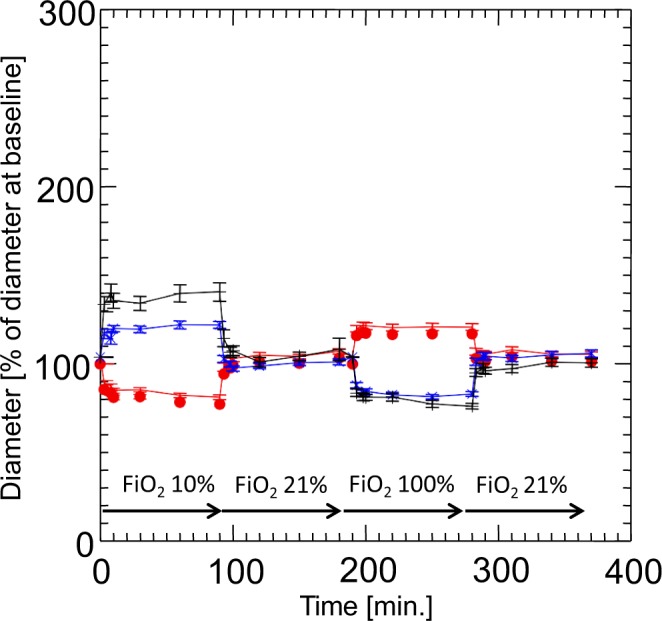
Oxygen-dependent diameters of microvessels (mean and SEM; n = 10 mice with a total of 23 lung and 12 endometrium grafts) of lung (filled circles), of endometrium (x), and of the native skinfold chamber microvessels (+). Measurements were performed at baseline (ambient air, i.e. FiO_2_ 20,97%; minute 0) during hypoxia (FiO_2_ 10%; minute 3–90), normoxia (ambient air, i.e. FiO_2_ 20.97%; min 91–180), and hyperoxia (FiO_2_ of 100%; minute 181–270) and normoxia (minute 270–360). Note that the lung vessels also at the ectopic transplant place in the skinfold chamber keep their origin-specific vasoreactivity to hypoxia, which is contrary to any other organ tissue of the body.

Mice subjected to hypoxia (FiO_2_ of 10%) showed a vasoconstriction by 23 ± 1% (mean ± SEM) in their lung graft vessels (Fig. 8). We furthermore observed a vasodilation by 17 ± 2% of the baseline (normoxia) vessel diameters under hyperoxia (Fig. 8). The observed vasoreactions were reversible under normalization of the inspired oxygen fraction and were comparable in all following assessments.

Contrary to the grafted lung tissue, the arterioles of the host striated muscle tissue within the dorsal skinfold chamber dilated by 36 ± 5% under hypoxia and constricted by 28 ± % when the animals were subjected to hyperoxia (Fig. 8). Endometrial microvessels showed a similar vasoreactivity pattern with a vasodilation by 18 ± 2% under hypoxia and a constriction by 21 ± 1% under hyperoxia. The extent of vasoconstriction/vasodilation at exposure to hypoxia (FiO_2_ of 10%) as a percent of baseline diameter did not differ in small and large graft vessels.

## Discussion

The repetitive *in vivo* analysis of ectopic lung tissue within mouse dorsal skinfold chambers turned out to be suitable to assess aspects of the observed microvasculature of lung tissue. We could observe angiogenesis occurring in the grafts on day 3 after transplantation, characterized by sinusoidal microvessels on the grafts’ edges with very slow centreline RBC velocity, perifocal oedema, and haemorrhage. After 10 days, the lung transplants were completely revascularized and exhibited a dense network of microvessels with irregular diameters, a chaotic angioarchitecture, and high centreline RBC velocity. Compared to lung tissue, the endometrium grafts contained a very structured, glomerulus-like vessel architecture with lower centreline RBC velocity. We could further show that despite missing ventilation, hypoxic vasoconstriction of the lung tissue arterioles occurred, whereas endometrium tissue arterioles dilated during hypoxia and constricted in hyperoxia.

A similar, lung graft skinfold chamber model to study neonatal tissue has already been established by *Sikora et al*. to study leukocyte-endothelial interactions as well as the reaction to the pleiotropic vasoconstrictor endothelin as well as to hypoxia^25,26^. In this setting, grafts were harvested from the neonatal lung of nude mice, and graft sizes were at least about 4 times larger, i.e. between 1 and 5 mm3. A huger graft size may have influenced the revascularization process and viability of the grafts. Vasoreactivity measurements were performed uniquely immediately after, but not during exposure to hypoxia. Hence, acute vasoreactivity could not be observed.

Specific to peripheral lung tissue are its widely distributed capillary network aimed at the alveolar gas exchange, thus its extremely rich, sponge-like vessel distribution. The normal pulmonary microvascular bed is of very low resistance and thus highly efficient concerning vascular throughput, as it has a huge reserve for the situation of increased cardiac output. One main finding of the current study was that lung tissue was slower revascularized than endometrial tissue. Furthermore, high vascular flow was observed in the lung grafts. Indeed, the centreline RBC velocity in lung grafts in those experiments was the highest so far observed by us in the revascularized tissue of dorsal skinfold chamber experiments.

Revascularization in terms of arterialization and venulization could not be predicted by us: we observed flow reversals as e.g. described in Fig. 1, pointing out that blood in-flow towards the graft and outflow out of the graft could change over time during the vascular integration of the graft. In contrast to endometrium grafts, the lung tissue grafting was associated with extensive peri-graft bleeding. Whether this was real bleeding as a coincidental finding, an indicator for frail vascular precursors, or a real prerequisite, e.g. as a basic step of an e.g. sinusoidal-like organization of vascularization as seen around day 3 post-transplantation and described in Fig. 3, remains open to us. This perigraft vascularization was observed both *in vivo* and assessed histologically: In histological slices, we found rather huge vessels with diameters of up to 70 µm, which was much more than the tiny vessels to be found around endometrium grafts that measured only about 21 µm.

Hypoxic pulmonary vasoconstriction is considered an essential mechanism that adapts lung perfusion to alveolar ventilation. It aims at shifting blood flow from hypoxic areas to normoxic ones. Its role is important e.g. in the fetal-neonatal transition, in the minute regulation of pulmonary gas exchange, in high-altitude pulmonary oedema, in acute lung injury and acute respiratory distress syndrome, as well as in pulmonary hypertension^27,28^. Some, but not all aspects of this sort of underlying oxygen sensing mechanism have been elucidated in different models and settings so that controversial concepts have not yet been unified. Sympathetic as well as parasympathetic nerves and also probably sensory nerves seem not to have an influence^28^.This suggests that, at least mechanistically, the amputated and therefore partially denervated, or partially nervally degenerated, and possibly also in part neurally re-ingrown lung tissue should not have led to any major modification, if neural structures were without any influence. Besides Kv-channels and L-type Ca2+ channels, cGMP via H_2_O_2_, and ATP as a potential second messenger, NADPH oxidases play prominent roles in the very acute phase of hypoxic pulmonary vasoconstriction, whereas mitochondria may play a role in sustained hypoxic pulmonary vasoconstriction^29^.

Rather surprisingly, in the dorsal skinfold chamber where lung tissue is vascularized like most other organs in a non-serial condition, the lung tissue maintains its response to whole animal hypoxia and hyperoxia “organ-specifically”. This has already been described by Sikora and the group of Sriramarao^25,26^. Their experimental setting contrasted, as their measurements have always been performed immediately after an experimental exposure. We could microscopically assess vasoreaction over time during the different inspiratory oxygen levels to the animal, as shown in Fig. 8. The finding of the preserved hypoxic vasoconstriction in the transplanted lung tissue points to an inherent regulation process of pulmonary vasoconstriction, which is at least in part independent of respiratory flow. Probably both, respiratory flow and peri-pulmonary innervation, seem therefore not critical for this lung-specific vascular regulation.

In conclusion, the described dorsal skinfold chamber lung transplant model using adult lung tissue allows the visualization of lung microvessels by epi-illumination vascular microscopy over time over a few weeks. Despite very few, and frequently virtually no visible inflammation observed within the graft, many questions are unanswered, such as the number of background mediators of inflammation, angiogenesis or fibrosis in the whole process and their consecutive effect to tiny regulations. Bearing those shortcomings in mind, *in vivo*-lung isograft microscopy may be suitable to study regulations of vascular or cellular vascular processes in the healthy lung as shown here. Also transplanted diseased lung or transplanted lung that is injured post-transplantation can be studied (not shown). The model allows the use of specific tissue or cells and tools that are unique to the variety of mouse genetic background.

## Materials and Methods

### Animals

The animal experiments have been approved by the according committee at the regional board for animal experiments of Homburg/Saar. All experiments were performed in conformity with the guiding principles for research involving animals and were approved by the German legislation on protection of animals. C57BL/6 mice (12–16 weeks old; body weight 20–30 g) of either sex were used. Mice were housed in single cages at a room temperature of 22–24 °C and a relative humidity of 60–65% with a 12-hour day/night cycle and had free access to tap water and standard laboratory chow (Altromin, Lage, Germany).

### Experimental model

**Figure 9:**
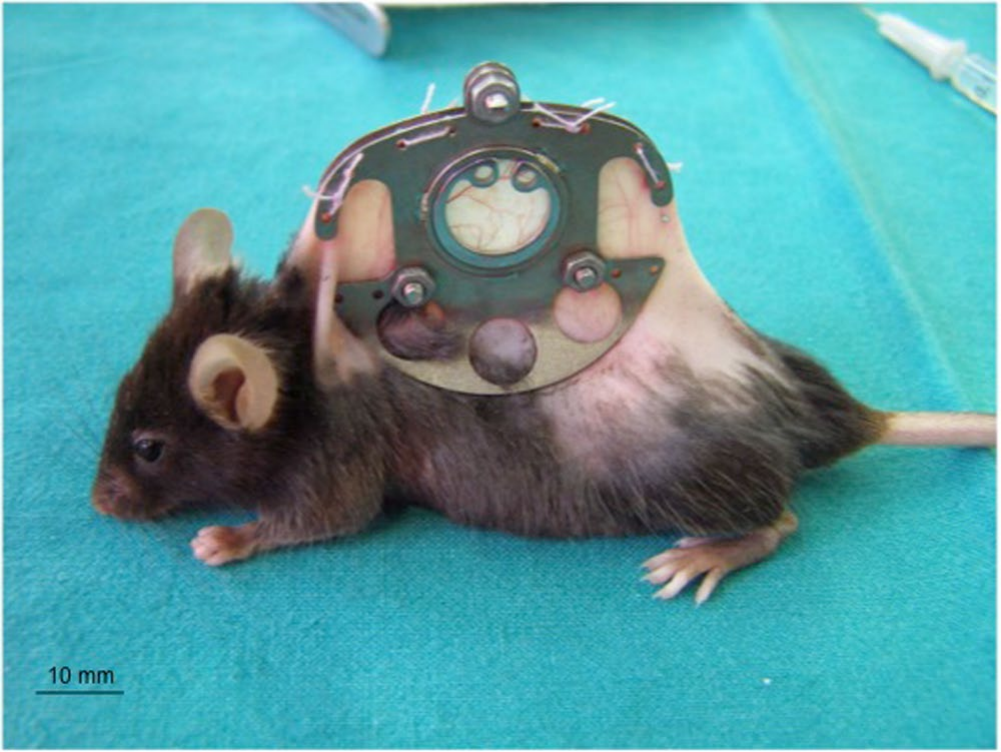
A C57BL/6 mouse carrying a dorsal skinfold chamber.

The dorsal skinfold chamber in mice was used for intravital fluorescence microscopy, as described previously in detail^30^. In brief, mice were anaesthetized intraperitoneally with a mixture of ketamine (75 mg/kg body weight; Merial GmbH, Hallbergmoos, Germany) and xylazine (20 mg/kg body weight; Ceva, Düsseldorf, Germany), and two symmetrical titanium frames were implanted to sandwich an extended double layer of the dorsal skin. One layer was completely removed in a 15-mm diameter circular area. The remaining layer consisting of the epidermis, subcutaneous tissue, and striated skin muscle was covered with a glass coverslip incorporated in one of the titanium frames. The animals tolerated the chamber well and showed no signs of discomfort or changes in sleeping and feeding habits (Fig. 9). A recovery period of 3 days was allowed before tissue transplantation and intravital observation.

### Lung isograft preparation and transplantation

Syngeneic donor mice were anaesthetized intraperitoneally as mentioned above. After sternotomy, lungs were excised and placed in a 30-mm diameter Falcon plastic Petri dish filled with 37 °C warm Dulbecco’s modified Eagle’s medium (DMEM; 10% fetal bovine serum, 0.1 mg/mL gentamicin) and the fluorescent dye bisbenzimide (H33342; 200 µg/mL; Sigma-Aldrich, Taufkirchen, Germany). The specific fluorescence/background fluorescence ratio is high enough to precisely delineate the stained lung tissue from the non-stained surrounding host tissue after transplantation into the dorsal skinfold chamber^31^. In addition, bisbenzimide, which stains the nuclear structure of the cells, can be used to study apoptotic cell death *in vivo* by analysing condensation, fragmentation, and margination of chromatin^32^. After carefully removing the pleura, subpleural lung tissue cubes of 500 µm diameter were prepared and transferred into 37 °C warm bisbenzimide-free DMEM. For isogenic transplantation of lung tissue, the cover glass of the dorsal skinfold chamber was removed, and three to four lung tissue cubes were placed on the striated muscle tissue within the chamber.

### Isolation and transplantation of endometrium grafts

For isolation of endometrium grafts, female mice equipped with a dorsal skinfold chamber were anaesthetized as mentioned above. After laparotomy, one uterus horn was aseptically removed and placed in a 30-mm diameter Falcon plastic Petri dish filled with 37 °C warm DMEM (10% fetal calf serum, 0.1 mg/mL gentamicin) and the fluorescent dye bisbenzimide (200 µg/mL). The uterus horn was opened longitudinally, and the endometrium was dissected from the uterine muscle under a stereomicroscope. Then, the endometrium was transferred into 37 °C warm bisbenzimide-free DMEM and microdissected into endometrium grafts of 500 µm diameter. For autologous transplantation of endometrium grafts, the cover glass of the dorsal skinfold chamber of the identical mouse was removed and one to two endometrium grafts were placed on the striated muscle tissue within the chamber together with one to two isogenic lung tissue cubes prepared as described above.

### Intravital epi-illumination fluorescence microscopy

Mice were anaesthetized and fixed on a Plexiglas platform that was placed on the microscopic stage. By use of a modified fluorescence microscope with a 100 W HBO mercury lamp (Axiotech vario, Zeiss, Göttingen, Germany), attached to an ultraviolet (330–380/>415 nm excitation/emission wavelength), blue (450–490/>515 nm) and green (525–555/>580 nm) filter system, the striated muscle and implanted tissue grafts´ microcirculation were analysed in epi-illumination technique. The microscopic images were recorded by a charge-coupled device (CCD) video camera (FK 6990-IQ, Pieper, Schwerte, Germany), transferred to a video system (S-VHS Panasonic AG 7350, Matsushita, Osaka, Japan), and recorded on videotape for subsequent off-line evaluation. Animals received an intravenous injection of 0.05 mL of 5% fluorescein isothiocyanate-labeled dextran 150.000 (Sigma-Aldrich) and 0.03 mL of rhodamine-6G (2 mg/mL; Sigma-Aldrich) into the retrobulbar plexus for vascular contrast enhancement and leukocyte staining *in vivo*. This allowed us to quantitatively assess arteriolar, capillary and venular perfusion as well as leukocyte flow and leukocyte-endothelial cell interactions^33^. Nuclei of tissue cells were visualized *in vivo* by topical application of 0.05 ml of bisbenzimide H33342 (0.01 mg/ml saline; Sigma)^34^ as described. Using ×5, ×10, ×20 long distance and ×62 water immersion objectives (Zeiss, Jena, Germany), magnifications of ×108, ×216, ×432 and ×1302 were achieved on a 14-inch video screen (PVM 1444, Sony, Tokyo, Japan). Furthermore, to determine the vitality of ectopic transplants, we qualitatively assessed the rate of apoptosis. Apoptosis is characterized by an increased nuclear condensation and fragmentation of chromatin.

### Microcirculatory and flow direction analysis

In order to set a clear baseline, the tissue grafts were scanned at ×108 or ×216 magnification on day 0 for measurement of graft size. At the subsequent observation time points (day 3, 6, 10, 14), additionally to measurement of graft size, 3 regions of interest per graft were first defined and furthermore repeatedly scanned at ×432 magnification. Quantitative off-line analysis of the videotapes was performed by means of the computer-assisted image analysis system CapImage (Zeintl, Heidelberg, Germany) and included the determination of the size of the transplanted lung and endometrium grafts (mm^2^, ×108 magnification), the size of the blood-perfused microvascular networks (given in percentage of the size of the grafts, ×108 magnification), the functional capillary density, i.e. the length of RBC-perfused microvessels per observation area (cm/cm^2^, ×432 magnification), the diameters of the microvessels (µm, ×432 magnification) and the centreline RBC velocity V_RBC_ (µm/s, ×432 magnification).

To assess recruitment of leukocytes to the graft, the number of adherent leukocytes (defined as cells that adhered to the microvascular endothelium over a period of >20 sec) was evaluated at ×432 magnification and divided by the functional capillary density of the individual ROIs. Numbers are given as cells/cm.

### Evaluation of tissue graft revascularization

After implantation of the lung and endometrium tissue grafts, the macroscopic appearance of the skinfold chamber preparation was controlled daily. In 15 mice with a total of 34 analysable lung transplants and 12 endometrium grafts (7 mice with 2–4 lung grafts, 8 mice with 1–2 lung grafts and 1–2 endometrium grafts), intravital fluorescence microscopic analyses were performed on days 0 (day of transplantation), 3, 6, 10 and 14 after transplantation. Functional capillary density was measured within three areas of interest per graft and observation time point. Microvessel diameters and microhemodynamic parameters were determined by analysing 10 microvessels per region of interest. Microvessels were selected randomly inasmuch as those microvessels were chosen, which crossed a vertical line drawn over the center of the video screen. At the end of the *in vivo* experiments, i.e. day 14 after transplantation of lung and endometrium grafts, the animals were euthanized with an overdose of pentobarbital (Merck, Darmstadt, Germany), and the dorsal skinfold chamber preparations were processed for haematoxylin and eosin staining and immunohistochemistry.

### Evaluation of microvascular vasoreactivity on modulated inspired oxygen fraction (hypoxia with FiO_2_ 10%; normoxia; hyperoxia with FiO_2_ 100%)

In 10 mice with a total of 23 lung isografts and 12 endometrium transplants (4 mice with 3 lung grafts, 6 mice with 1–2 lung grafts and 2 endometrium grafts), vasoreactivity on different oxygen fractions over time was tested. Between day 10 and 13 after transplantation, mice were anaesthetized and fixed on a Plexiglas platform placed on the microscopic stage. After injection of 0.05 mL of 5% fluorescein isothiocyanate-labeled dextran 150.000, diameters of ~5 arterioles of the striated muscle tissue within the chamber and 5 arterioles per graft were determined while the animals were breathing room air. Video printouts were made during videography and initially marked to indicate the exact localization for the measurement of vessel diameters. Afterwards, mice were exposed to either hypoxia (FiO_2_ of 10%) or hyperoxia (FiO_2_ of 100%) using a face mask during 90 min and then, to normoxia for 90 min on the first day and the other way around on the next day. Vessel diameters were measured at baseline and at 3, 8, 10, 30, 60 and 90 min of each FiO_2_.

### Histology and immunohistochemistry

For light microscopy, the chamber tissue was excised, fixed in 4% phosphate-buffered formalin for 3 days and embedded in paraffin. Four-µm thick sections were cut and stained with haematoxylin HE and eosin according to standard procedures. For immunohistochemical detection of smooth muscle lining the wall of the blood vessels within the grafts, α-SMA staining was performed with a mouse monoclonal anti-SMA antibody as primary antibody (1:100; Sigma-Aldrich, Taufkirchen, Germany). A goat anti-mouse horse reddish peroxidase (HRP) antibody (1:200; Dianova, Hamburg, Germany) served as secondary antibody. 3, 3′-Diaminobenzidine was used as chromogen. The sections were counterstained with hemalaun and examined by light microscopy (BX60; Olympus, Hamburg, Germany).

Apoptotic cells within the tissue samples were visualized immunohistochemically in 4 mice using a polyclonal rabbit anti-cleaved caspase-3 antibody (1:100; New England Biolabs, Frankfurt, Germany) as primary antibody, staining activated caspase-3. A goat anti-rabbit biotin-marked antibody (1:100, Dianova, Hamburg, Germany) served as secondary antibody. 3, 3′-Diaminobenzidine was used as chromogen. The sections were counterstained with hemalaun and examined by light microscopy (BX60; Olympus, Hamburg, Germany). Each section (4–8 per mouse) was divided into high power fields dependent on graft size (4–30/section for graft, 3–21/section for skin striated muscle). Data are given as apoptotic cells per high power field.

### Statistics

Data were first analysed for normal distribution and equal variance. Differences between groups were then calculated by analysis of variance (ANOVA) followed by the appropriate post hoc test (Fisher’s LSD) compensating for multiple comparisons. To test for time effects within each experimental group, ANOVA for repeated measurements was applied. This was followed by a type I error Bonferroni-corrected post hoc paired comparison for repeated measurements (SigmaStat; Jandel Corporation, San Rafael, CA, USA). All data are given as means ± standard error of the mean (SEM). Statistical significance was accepted for a value of P < 0.05.
